# Insights from molecular docking and molecular dynamics on the potential of vitexin as an antagonist candidate against lipopolysaccharide (LPS) for microglial activation in neuroinflammation

**DOI:** 10.1186/s12896-021-00697-4

**Published:** 2021-06-05

**Authors:** M. A. F. Yahaya, A. R. Abu Bakar, J. Stanslas, N. Nordin, M. Zainol, M. Z. Mehat

**Affiliations:** 1 Department of Human Anatomy, Faculty of Medicine & Health Sciences, Universiti Putra Malaysia (UPM), 43400 Serdang, Selangor Malaysia; 2 Department of Medicine, Faculty of Medicine & Health Sciences, Universiti Putra Malaysia (UPM), 43400 Serdang, Selangor Malaysia; 3 Department of Obstetrics & Gynaecology, Faculty of Medicine & Health Sciences, Universiti Putra Malaysia (UPM), 43400 Serdang, Selangor Malaysia; 4 Bioassay Unit, Herbal Medicine Research Centre (HMRC), Institute for Medical Research (IMR), National Institute of Health (NIH), Jalan Setia Murni U13/52, Seksyen U13, Bandar Setia Alam, 40170 Shah Alam, Selangor Malaysia; 5 Department of Chemical Engineering Technology, Faculty of Engineering Technology, Universiti Malaysia Perlis (UniMAP), 01000 Kangar, Perlis Malaysia

**Keywords:** Vitexin, Molecular docking, Molecular dynamics, Antagonist, Neuroinflammation, Microglial cell

## Abstract

**Background:**

Neuroinflammation has been identified to be the key player in most neurodegenerative diseases. If neuroinflammation is left to be unresolved, chronic neuroinflammation will be establish. Such situation is due to the overly-activated microglia which have the tendency to secrete an abundance amount of pro-inflammatory cytokines into the neuron microenvironment. The abundance of pro-inflammatory cytokines will later cause toxic and death to neurons. Toll-like receptor 4 (TLR4)/MD-2 complex found on the cell surface of microglia is responsible for the attachment of LPS and activation of nuclear factor-κB (NF-κB) downstream signalling pathway. Albeit vitexin has been shown to possess anti-inflammatory property, however, little is known on its ability to bind at the binding site of TLR4/MD-2 complex of microglia as well as to be an antagonist for LPS.

**Results:**

The present study reveals that both vitexin and donepezil are able to bind at the close proximity of LPS binding site located at the TLR4/MD-2 complex with the binding energy of − 4.35 and − 9.14 kcal/mol, respectively. During molecular dynamic simulations, both vitexin and donepezil formed stable complex with TLR4/MD-2 throughout the 100 ns time length with the root mean square deviation (RMSD) values of 2.5 Å and 4.0 Å, respectively. The root mean square fluctuation (RMSF) reveals that both compounds are stable. Interestingly, the radius of gyration (rGyr) for donepezil shows notable fluctuations when compare with vitexin. The MM-GBSA results showed that vitexin has higher binding energy in comparison with donepezil.

**Conclusions:**

Taken together, the findings suggest that vitexin is able to bind at the binding site of TLR4/MD-2 complex with more stability than donepezil throughout the course of 100 ns simulation. Hence, vitexin has the potential to be an antagonist candidate for LPS.

## Introduction

Neuroinflammation has been postulated by many to be the key player in most neurodegenerative diseases [1, 2]. Examples of neurodegenerative diseases are Alzheimer’s disease (AD) and Parkinson’s disease (PD). In the U. S, it is estimated that 13.8 million people will be suffering from AD by 2050 [3]. On the other hand, the prevalence of the disease in Malaysia is estimated to reach 0.454% by 2050 [4].

Currently, there are only five drugs that have been approved by the U.S. Food & Drug Administration (FDA) for the treatment of neurodegenerative diseases. One of the drugs is donepezil. Donepezil has been clinically used as part of AD treatment regime due to its ability to act as a potent anti-inflammatory agent as [5, 6]. In addition, donepezil has been reported to be able to deactivate microglia independently of its acetylcholine receptor [7]. However, the consumption of donepezil only able to delay the progression of AD but not curing the disease [8].

Upon the establishment of neuroinflammation, microglia are said to be among the first cells to be activated [9, 10]. The activation of microglia allows the damage to be repaired in a short period of time to maintain the homeostasis of neuron microenvironment. However, microglia can become dysregulated when the repairing process takes longer time. Such situation results in the establishment of chronic neuroinflammation [11].

In chronic neuroinflammation, the overly-activated microglial cells have been identified to be the culprit in the progression of neurodegenerative diseases [12, 13]. The overly-activated microglial cells have the tendency to excessively secrete a myriad of pro-inflammatory cytokines (e.g. interleukin (IL)-6, IL-1β and tumour necrosis factor-α (TNF-α)) upon triggered with its stimuli such as lipopolysaccharide (LPS).

The LPS will interact with TLR4/MD-2 complex found on the cell surface of microglial cells [14–16]. The attachment of LPS at the binding site of TLR4/MD-2 complex allows the induction of downstream signalling cascade [17, 18]. This phenomenon will cause the activation of nuclear factor-κB (NF-κB) transcription factor that subsequently express the pro-inflammatory cytokines [19].

The involvement of TLR4/MD-2 complex in neuroinflammation has been reported in recent years [20–22]. TLR4/MD-2 complex is linked with memory deficit in the presence of Aβ oligomers (Aβo) [23]. The presence of Aβo allows the increase level of pro-inflammatory cytokines secreted by the activated microglia [23]. On the other hand, Miron et al. (2018) reported the increase level of TNF-α and IL-6 genes expression when the authors conducted the gene profile analysis of post-mortem human brains suffering from AD [24]. Therefore, inhibiting the TLR4 signalling pathway has been proposed to be an effective therapeutic strategy to suppress the undesirable amount of pro-inflammatory cytokines [25].

Albeit a number of antagonist against TLR4/MD-2 complex has been developed and proceeded to clinical trials, however, none of these antagonists have shown a success in meeting the primary endpoint to reduce the patient’s mortality rate [26, 27]. Hence, the need to find a new antagonist against TLR4/MD-2 complex is much need.

Vitexin (apigenin-8-C-β-D-glucopyranoside) can be found in a number of medicinal plant species namely *Ficus deltoidea* [28], pearl millet [29], and bamboo [30] as one of the plants’ major active compounds. The compound also has been known to possess a number of pharmacological properties such as anti-inflammatory [31, 32] and neuroprotective effect [33]. In addition, vitexin has recently been explored on its potential to play a role in epigenetic activities [34]. Albeit numerous studies have shown its ability to act as anti-inflammation and neuroprotection, however, the information on the ability of the compound to bind at the LPS binding site on TLR4/MD-2 complex and hence acting as the antagonist for LPS is yet to be fully elucidated. Hence, the present study aimed to determine the ability of vitexin to bind at the binding site of TLR4/MD-2 complex, to determine the stability of vitexin-TLR4/MD-2 complex for the course of 100 ns and to determine the potential of vitexin to be an antagonist against LPS.

## Methodology

### Receptor and ligand preparation

The protein crystal structure of Toll-like receptor 4 (TLR4)/MD-2 complex (PDB ID: 3VQ2 with resolution of 2.48 Å [15]) was retrieved from Protein Data Bank (https://www.rcsb.org/).

The 3D structures of vitexin (PubChem CID: 5280441) and donepezil (PubChem CID: 5741) were retrieved from PubChem database in .sdf file format and were later converted into .pdb format via online (https://cactus.nci.nih.gov/translate/). Both ligands were optimised by using UCSF Chimera [35] to obtain the most stable 3D conformation.

### Molecular docking

The polar hydrogen and Kollman partial atomic charge were assigned to TLR4/MD-2 complex by using AutoDock4 software [36] and saved as AutoDock readable file. Both ligands (vitexin and donepezil) were made flexible, torsion root was set free and the protein was kept rigid. The protein binding site was defined at Leu54, Lys89, Arg90, Lys91, Lys122, Ile124, Lys125, Lys128, Tyr131 and Lys132 as described by [14, 15] with the grid size of 50 Å × 50 Å × 50 Å and spacing of 0.375 Å. The grid box was set at *x* = − 20.312, *y* = − 18.262, *z* = 23.949. Lamarckian genetic algorithm [37] was used in this process with the energy evaluation of 250,000 and a total of 100 runs inside the binding site. The outcome from this docking was later analysed by using AutoDockTools software [36]. The best docked scoring pose as determined by AutoDock software was selected and visualised by using Biovia Discovery Studio Visualizer.

### Ligand-receptor interaction analysis

The 2-dimensional (2D) and surface annotation of both ligand interactions with the protein were generated and analysed by using Biovia Discovery Studio Visualizer.

### Molecular dynamics (MD) simulations

MD simulations for 100 ns were carried out using Desmond Simulation Package (Schrödinger, LLC) [38]. The protein-ligand complexes were processed by the Protein Preparation Wizard Tool by using default parameters [39]. Transferable Intermolecular Interaction Potential 3 Points (TIP3P) has been selected as the solvent model with 10x10x10 Å orthorhombic box. The counter ions (Na^+^ or Cl^−^) were added and OPLS_2005 force field parameters were used [40]. The *NPT* ensemble with a temperature of 300 K as well as 1 atm pressure were applied during the simulations. To mimic the physiological conditions, 0.15 M of NaCl was added. The trajectories from the MD simulations were saved for every 50 ps intervals for analyses of root mean square deviation (RMSD), root mean square fluctuation (RMSF) as well as the protein-ligand contacts. The simulations were repeated thrice.

### Molecular mechanics-generalised born surface area (MM-GBSA) calculations

The binding free energy calculation of the protein-ligand docking complexes was estimated by using the Prime-MM/GBSA by using OPLS_2005 force field [41]. Prime MM-GBSA method calculates the binding free energy as follows:

$$ {\Delta  \mathrm{G}}_{\mathrm{binding}}={\mathrm{G}}_{\mathrm{docking}\ \mathrm{complex}}-\left({\mathrm{G}}_{\mathrm{protein}}+{\mathrm{G}}_{\mathrm{ligand}}\right) $$

Where, ∆G_binding_ = binding free energy, G_docking complex_, G_protein_, and G_ligand_ are the free energies of the docking complex, protein and ligand, respectively. The obtained results were presented as the mean ± standard deviation (SD).

## Result

### Molecular docking analysis

**Fig. 1:**
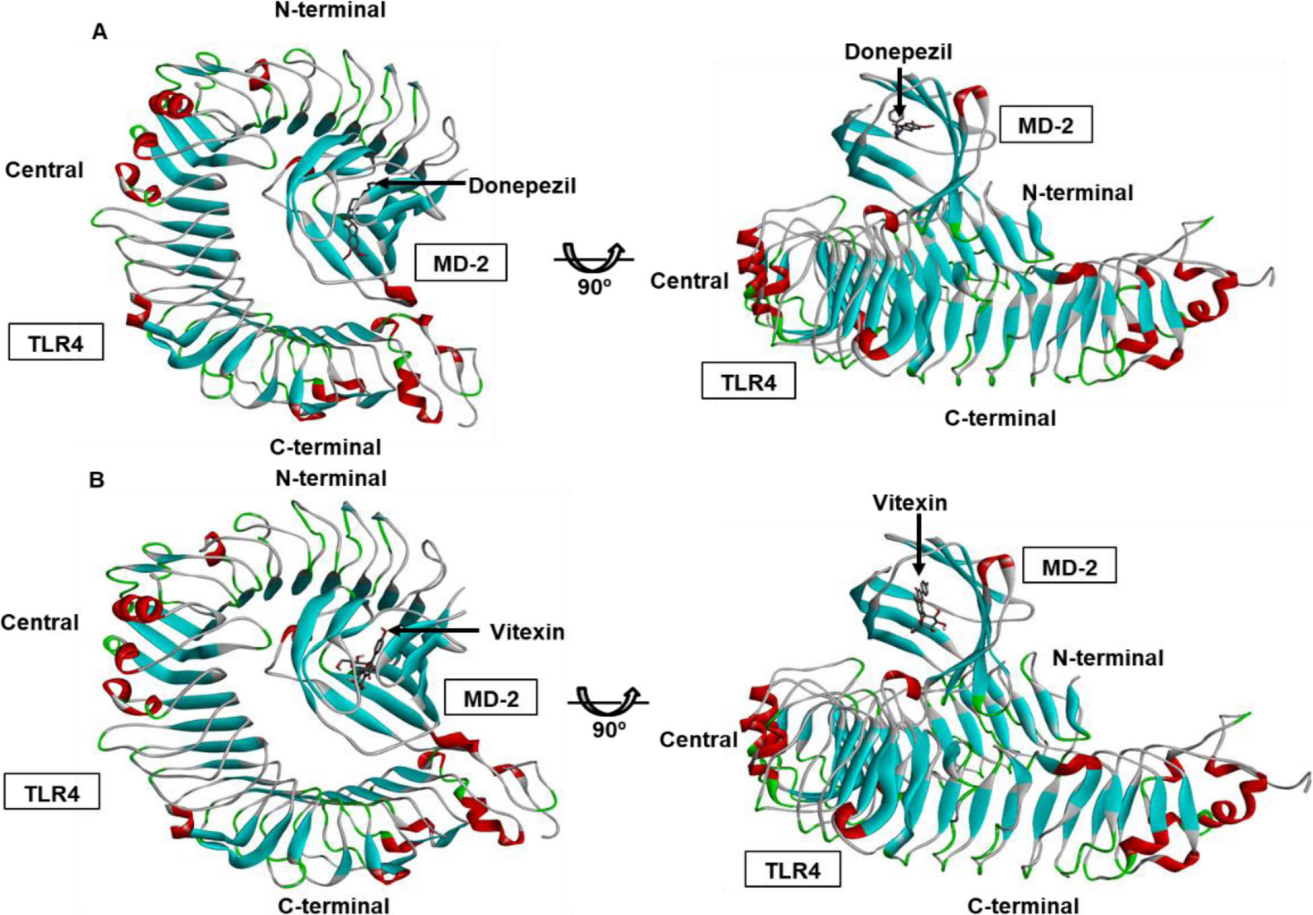
Molecular docking visualisation of (**A**) donepezil and (**B**) vitexin against TLR4/MD-2 complex by using Biovia Discovery Studio Visualizer. Both ligands docked at the binding pocket of the TLR4/MD-2 complex

The binding energy of vitexin and donepezil against TLR4/MD-2 complex was analysed by using AutoDockTools software [36]. The grid box was set at the protein binding site as previously described by [14, 15]. The docking results for both ligands were clustered with the RMSD tolerance of 2.0 Å. The best docked scoring pose as determined by AutoDock software was selected and visualised by using Biovia Discovery Studio Visualizer. The binding sites of both vitexin and donepezil on TLR4/MD-2 complex are shown in Fig. 1.

The AutoDockTools software generated the output file and log file for each complex. Donepezil binds to TLR4/MD-2 complex with the binding energy of − 9.14 kcal/mol. On the other hand, vitexin binds to TLR4/MD-2 complex with the binding energy of − 4.35 kcal/mol. The summary of the docking analysis for both donepezil and vitexin is listed in Table 1.

**Table 1:** Summary of docking analysis for donepezil and vitexin by using AutoDockTools software

Ligand	RMSD (Å)	Binding Energy (kcal/mol)	Inhibition Constant, Ki	Intermolecular Energy (kcal/mol)	Electrostatic Energy (kcal/mol)	Internal Energy (kcal/mol)	Torsion Free Energy (kcal/mol)	Unbound System’s Energy (kcal/mol)
Donepezil	37.35	−9.14	198.79 nM	−10.93	−0.27	− 0.89	1.79	− 0.89
Vitexin	35.59	−4.35	647.72 μM	−7.33	−0.02	−3.71	2.98	−3.71

### Ligand-receptor interaction analysis

**Fig. 2:**
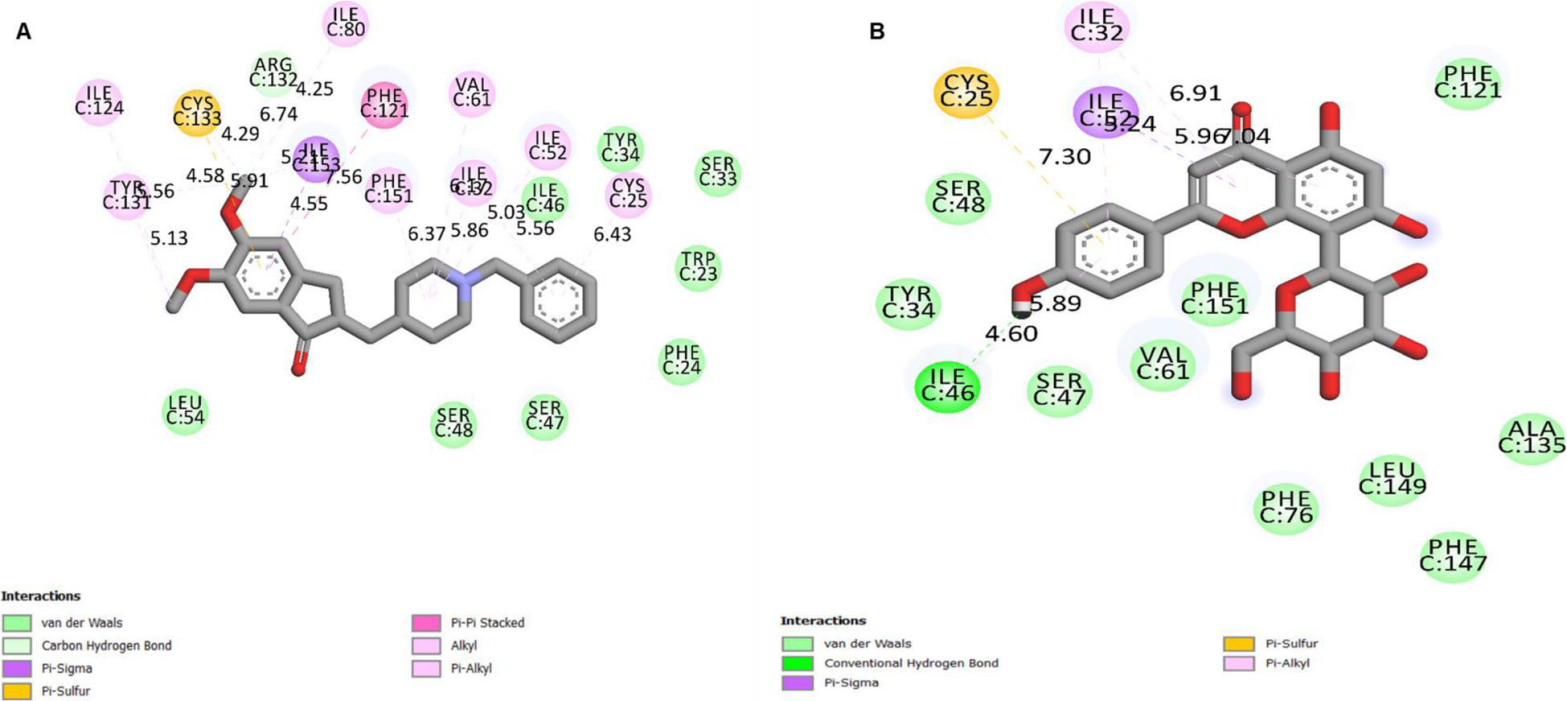
2D residues diagram analysis for (**A**) donepezil and (**B**) vitexin along with their respective bonds generated by Biovia Discovery Studio Visualizer

From Fig. 1, both ligands were able to dock at the binding pocket of TLR4/MD-2 complex. At the binding pocket of TLR4/MD-2 complex, both ligands interact with various number of amino acids with their respective interaction bond. As shown in Fig. 2, donepezil interacted with Cys25, Ile32, Ile52, Val61, Ile80, Phe121, Ile124, Tyr131, Arg132, Cys133, Phe151 and Ile153. On the other hand, vitexin is shown to interact with Cys25, Ile32, Ile46 and Ile52. The summary of their residues along with their respective bond distance (Å) and type of interacted bond is listed in Table 2.

**Table 2:** Summary of 2D residues diagram analysis for donepezil and vitexin generated by Biovia Discovery Studio Visualizer

Ligand	Interaction Amino Acid Residue	Bond Distance (Å)	Type of Interacted Bond
Donepezil	Cys25	6.43	π-Alkyl
	Ile32	5.56 and 5.86	π -Alkyl and Alkyl
	Ile52	5.03	Alkyl
	Val61	6.17	Alkyl
	Ile80	4.25	π -Sulfur
	Phe121	7.56	π - π stacked
	Ile124	5.56	Alkyl
	Tyr131	5.13 and 5.91	π -Alkyl and π -Alkyl
	Arg132	6.74	Carbon Hydrogen
	Cys133	4.29 and 4.58	Alkyl and π -Sulfur
	Phe151	6.37	π -Alkyl
	Ile153	4.55 and 5.21	π -Sigma and Alkyl
Vitexin	Cys25	7.30	π -Sulfur
	Ile32	5.24 and 6.91	π -Alkyl and π -Alkyl
	Ile46	4.60 and 5.89	Van der Waals and π -Alkyl
	Ile52	5.96 and 7.04	π -Sigma and π -Alkyl

### Molecular dynamics (MD) simulations

MD simulations were carried out for donepezil-TLR4/MD-2 complex and vitexin-TLR4/MD-2 complex at 100 ns by using Desmond Simulation Package. The simulations were performed for three times. RMSD plots illustrate the RMSD evolution of protein (left y-axis). All protein frames were first aligned on the reference frame backbone. Then, the RMSD was calculated based on the atom selection. By monitoring the protein RMSD, it allows the present study to determine its structural conformation during the simulation period.

On the other hand, the ligand RMSD (right y-axis) depicts the stability of the ligand with respect to the protein and its binding pocket. The ‘Lig fit Prot’ shows the RMSD of a ligand when the protein-ligand complex was first aligned at the protein backbone of the reference and then the RSMD of the ligand heavy atoms was measured.

**Fig. 3:**
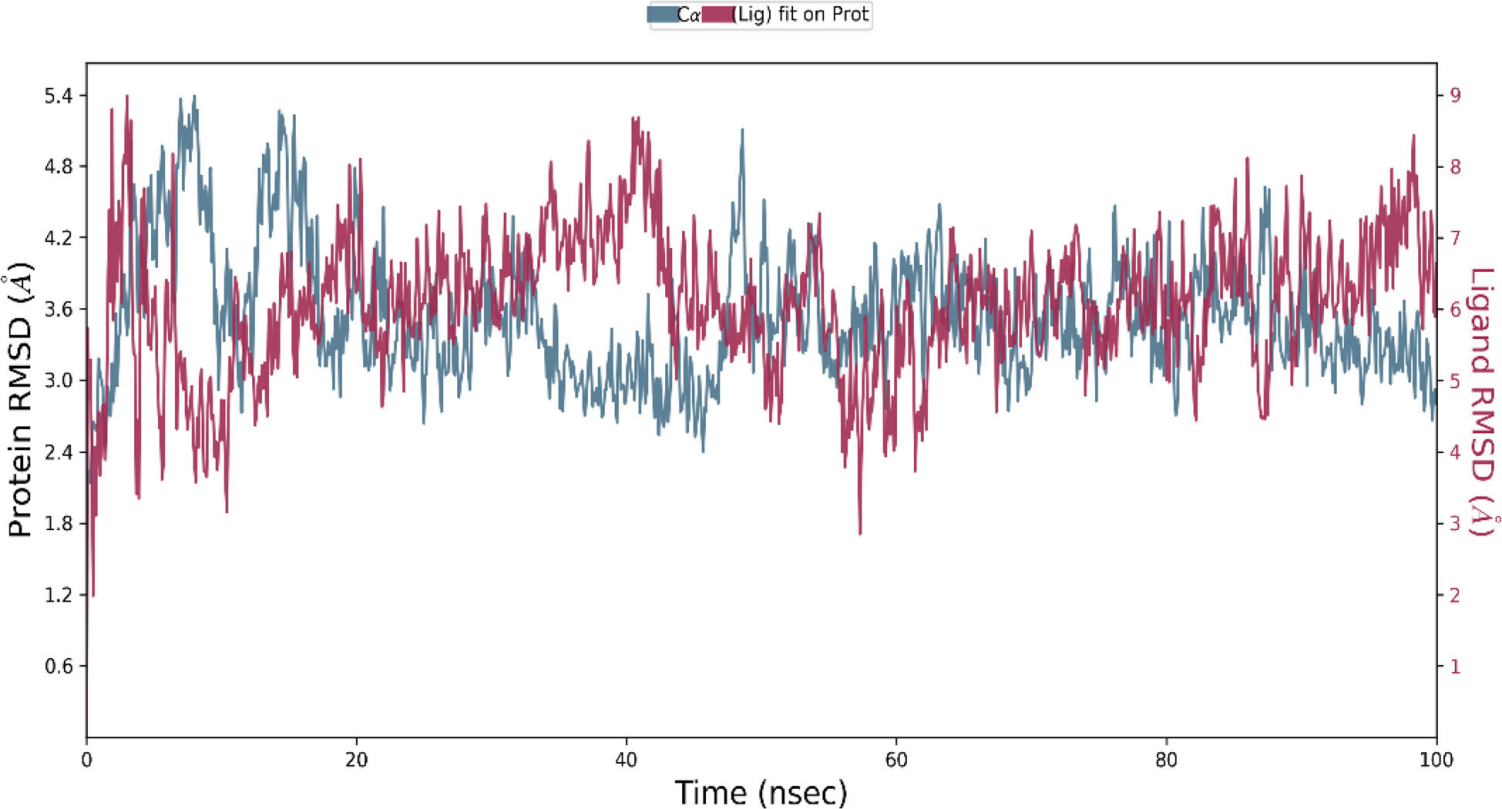
RMSD (**Å**) of the Cα atoms of TLR4/MD-2 complex and donepezil against time (nsec). The left y-axis shows the variation in the TLR4/MD-2 complex RMSD against time. The right y-axis shows the variation in the donepezil RMSD against time

The donepezil-TLR4/MD-2 complex plot (Fig. 3) shows that both donepezil and TLR4/MD-2 complex were stabilised after 65 ns at 4.0 Å for both protein and ligand. However, a slight deviation of protein structure was observed after 90 ns. As for the ligand, the deviation was observed at 85 ns. This plot shows that the protein structure managed to stabilise after 65 ns but the ligand underwent continuous conformational changes which suggests that the ligand might require longer simulation time in order for it to become stable.

**Fig. 4:**
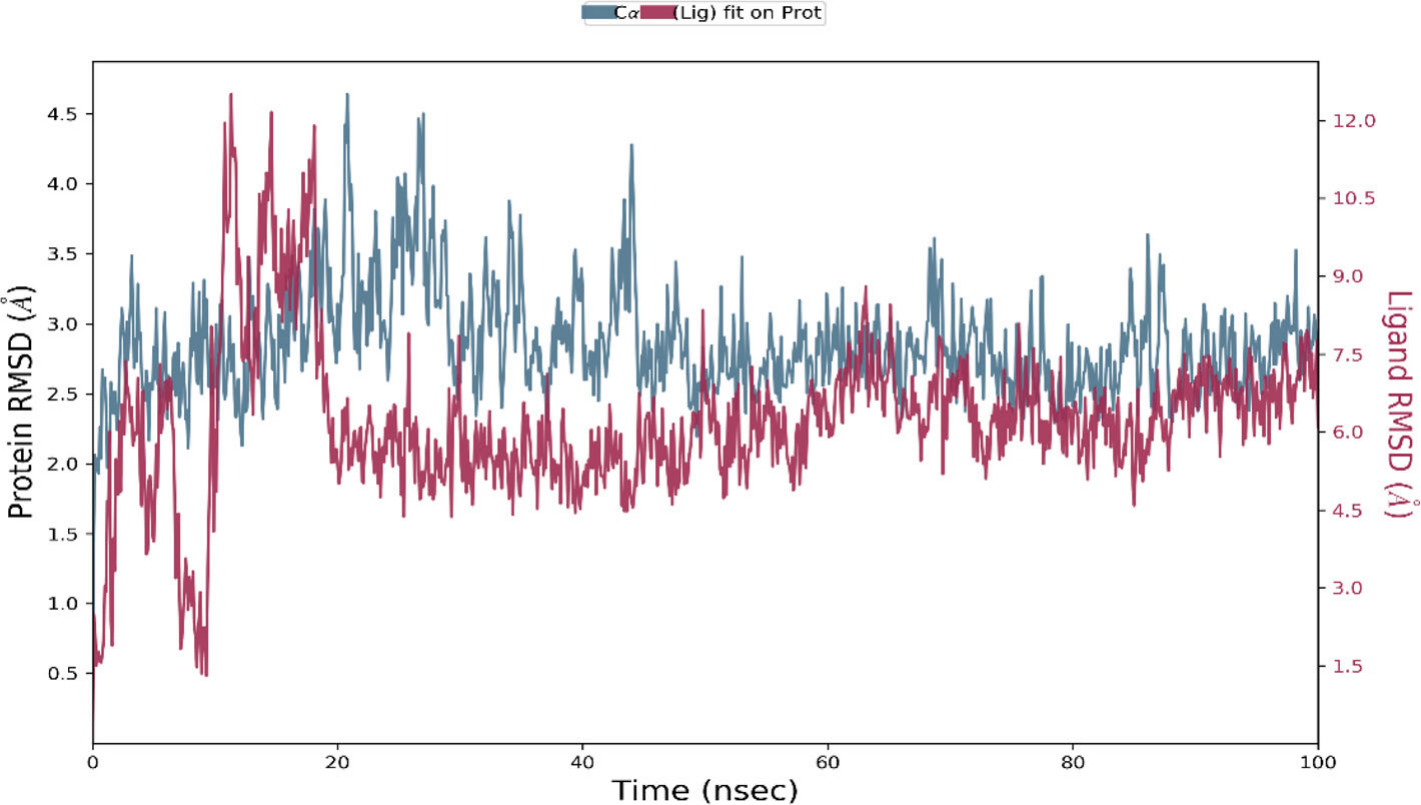
RMSD (**Å**) of the Cα atoms of TLR4/MD-2 complex and vitexin against time (nsec). The left y-axis shows the variation in the TLR4/MD-2 complex RMSD against time. The right y-axis shows the variation in the vitexin RMSD against time

As for vitexin-TLR4/MD-2 complex plot (Fig. 4), both vitexin and TLR4/MD-2 complex were stabilised after 20 ns at 2.5 Å for ligand and 4.0 Å for protein. The ligand was able to stabilise until 50 ns before it underwent a slight deviation after 60 ns. It can be observed that vitexin-TLR4/MD-2 complex structure was able to continuously interacting at the same deviation rate by showing less deviation in the structure. Vitexin-TLR4/MD-2 complex seemed to be able to stabilise at most of the time during simulation.

**Fig. 5:**
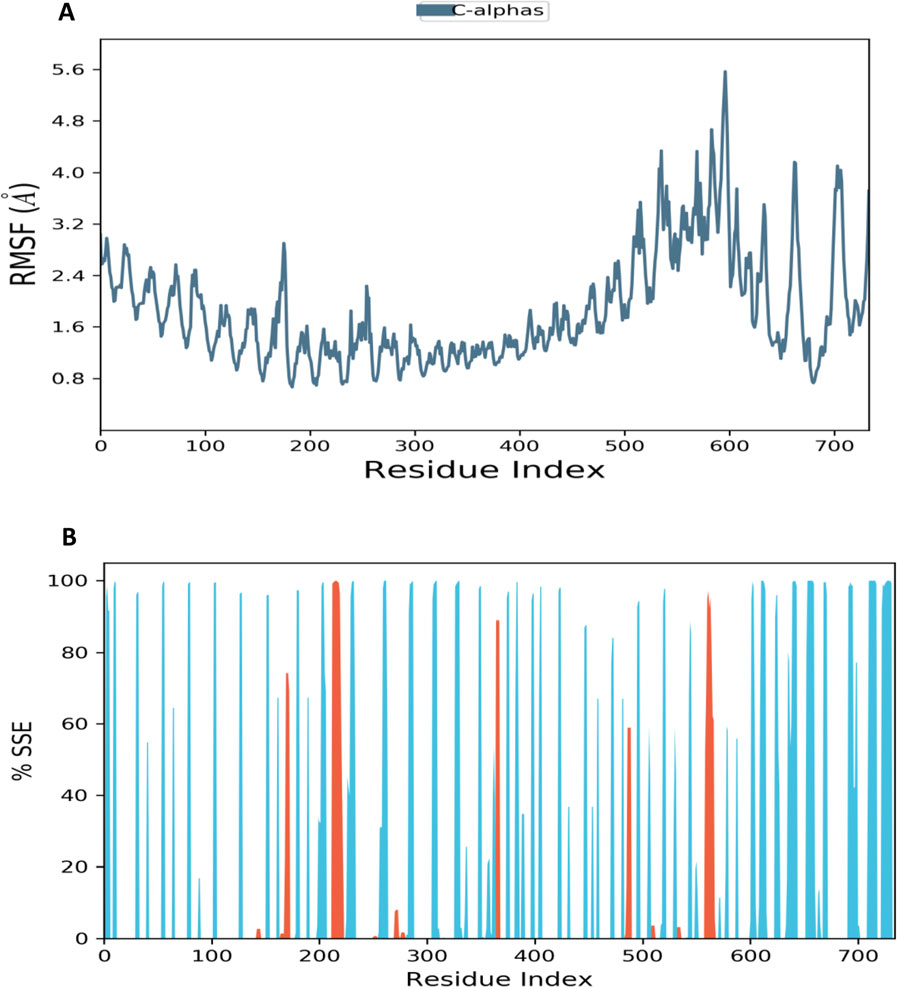
Analysis of (**A**) residue-wise RMSF and (**B**) protein secondary structure elements (SSE) of TLR4/MD-2 complex upon binding with donepezil. The red columns indicate alpha helices whereby blue columns indicate beta-strands

**Fig. 6:**
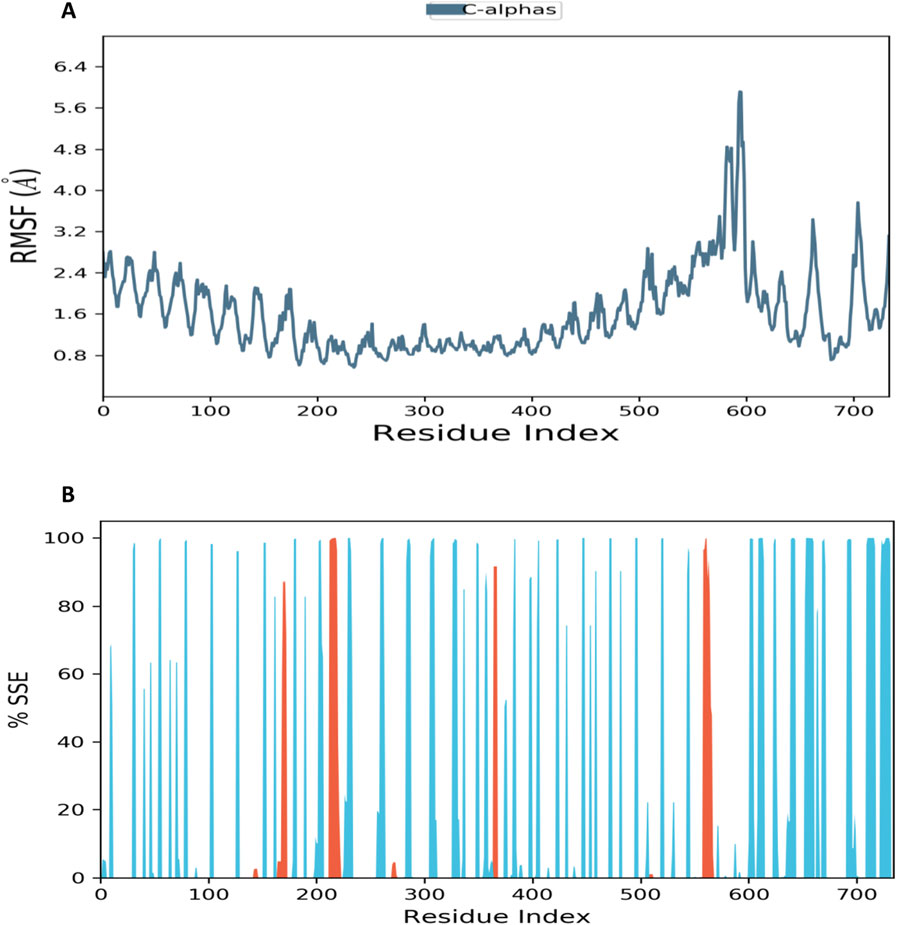
Analysis of (**A**) residue-wise RMSF and (**B**) protein secondary structure elements (SSE) of TLR4/MD-2 complex upon binding with vitexin. The red columns indicate alpha helices whereby blue columns indicate beta-strands

Figs. 5 and 6 show the analysis values of residue-wise RMSF and protein secondary structure element (SSE) when TLR4/MD-2 complex bound with donepezil and vitexin, respectively. Both Figs. 5 and 6 show almost similar pattern peaks in which the higher peaks correspond to loop regions identified from the MD simulation trajectories. The lower value of RMSF indicates the stability of ligands binding to TLR4/MD-2 complex. Protein secondary structure elements (SSE) analysis displayed the formation of more β-sheets (blue) as compared to α-helices (orange) in TLR4/MD-2 complexed with donepezil (Fig. 5B). While comparing with RMSF plot, it was observed that significant amino acid fluctuations at the respective positions having larger fluctuations (Fig. 5A) conform into less stable α-helices (orange) corroborated with the SSE analysis. Similar pattern was also observed in the TLR4/MD-2 and vitexin bound complex (Fig. 6A and B).

**Fig. 7:**
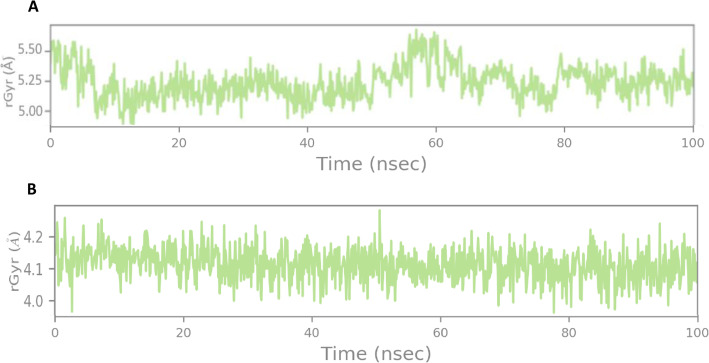
Radius of gyration (rGyr) analysis for (**A**) donepezil and (**B**) vitexin with respect to TLR4/MD-2 complex. Donepezil shows more fluctuations at higher Å compared to vitexin

Radius of gyration (rGyr) is used as an indicator to determine the compactness of protein structure [42]. Figure 7 shows the rGyr plot for Cα atoms and protein throughout the course of 100 ns simulation. It can be observed that donepezil shows a notable fluctuation in comparison with vitexin. This indicates that donepezil might have undergone a significant structural transition compared to vitexin.

### MM-GBSA calculations

Utilizing the MD simulation trajectory, the binding free energy along with other contributing energy in form of MM-GBSA were determined for donepezil and vitexin with TLR4/MD-2 complex. The results (Table 3) suggested that the maximum contribution to ΔG_bind_ in the stability of the simulated complexes were due to ΔG_bind_Coulomb, ΔG_bind_vdW and ΔG_bind_Lipo. In contrast, ΔG_bind_Covalent and ΔG_bind_SolvGB energies contributed to the instability of the corresponding complexes. The binding energy was found higher in vitexin bound complex having dG = − 73.109 ± 8.4 kcal/mol as compared to donepezil bound complex with TLR4/MD-2 complex (Table 3). Therefore, MM-GBSA outcome suggested that the vitexin has higher potential as antagonist against TLR4/MD-2 complex in comparison with donepezil and the efficiency of the drugs in binding to the selected protein and the ability to form stable protein-ligand complexes.

**Table 3:** Binding free energy components for the docking complexes of TLR4/MD-2 protein with donepezil and vitexin calculated by MM-GBSA analysis

Compound	MM-GBSA (kcal/mol)					
	ΔG_**bind**_	ΔG_**bind**_Lipo	ΔG_**bind**_vdW	ΔG_**bind**_Coulomb	ΔG_**bind**_SolvGB	ΔG_**bind**_Covalent
Donepezil	−54.201 ± 6.3	−24.94 ± 1.2	−28.61 ± 2.7	− 19.49 ± 5.4	19.44 ± 2.8	0.96 ± 0.6
Vitexin	− 73.109 ± 8.4	− 33.56 ± 2.0	− 45.32 ± 6.7	− 45.038 ± 8.9	49.30 ± 7.42	2.57 ± 1.2

The MM-GBSA final trajectory of 100 ns simulations of vitexin and donepezil bound to TLR4/MD-2 exhibited a stabilized and converged after simulation. Due to arrangement of the ligands at the binding site during MD simulation resulted in high binding energy and a stabilized complex.

## Discussion

Microglia have become the subject of interest amongst researchers since the cells have been shown to be one of the major culprits in neurodegenerative diseases. Microglia has the ability to act as an enhancer for neuroinflammation which eventually lead to the death of neurons [43]. In its normal state, microglia have the role in maintaining the homeostasis of neuron microenvironment, influencing the brain development and respond towards any injury [44]. For the latter, microglia need to be stimulated by its stimuli such as LPS in order for the cells to be in their active state [45].

However, when microglia become overly-activated in chronic neuroinflammation condition, the cells have the tendency to become dysregulated by excessively produce higher amount of pro-inflammatory cytokines (e.g. tumour necrosis factor-α (TNF-α), interleukin-6 (IL-6)) into its microenvironment [46]. This excessive amount of pro-inflammatory cytokines will cause toxic to the neurons and ultimately encourage the progression of neuroinflammation.

Kim et al. (2007) and Ohto et al. (2012) have shown that the TLR4/MD-2 complex found on the surface of microglial cells is crucial for the recognition of LPS [14, 15]. The activation of microglia by LPS through TLR4/MD-2 complex has allowed the induction of the downstream neuroinflammatory pathways such as nuclear factor-κB (NF-κB) signalling pathway [45, 47]. As a result, pro-inflammatory cytokines will be secreted and thus, contribute to the worsen of neurodegenerative diseases.

In light of the therapeutic strategy to block the activation of TLR4 which gives rise to the chronic inflammation [48], the present study has chosen vitexin due to its reported anti-inflammatory and neuroprotective properties [31–33]. Conversely, the U.S. Food & Drug Administration (FDA)-approved AD drug; donepezil, has been selected to compare the its efficacy with vitexin in acting as antagonist against TLR4/MD-2 complex of microglia. Donepezil has been shown to not only able to inhibit the cholinergic activity, the drug also revealed to have potent anti-inflammatory effects in AD patients as well as in LPS-treated animals [5, 6]. Furthermore, Hwang et al. (2010) reported that donepezil managed to deactivate microglia independently of its acetylcholine (ACh) receptor [7]. However, Hwang et al. (2010) did not report that the deactivation of microglia was due to the interaction of donepezil with TLR4/MD-2 complex [7]. To the best of our literature search, the present study is the first in silico study to be reporting the predictive ability of donepezil to bind at the binding site of TLR4/MD-2 complex.

In reference to [14, 15] studies, they have reported that Leu54, Lys89, Arg90, Lys91, Lys122, Ile124, Lys125, Lys128, Tyr131 and Lys132 are the essential site for the LPS to bind in order for the microglia to be activated. Hence, in preparing for the molecular docking analysis, this binding site has been covered during the grid box setting. The results from this study show that both donepezil and vitexin are able to bind at the binding pocket of TLR4/MD-2 complex with the binding energy of − 9.14 kcal/mol and − 4.35 kcal/mol, respectively.

Albeit vitexin did not bind at the exact binding site of LPS at TLR4/MD-2 complex as mentioned by [14, 15], however, the compound bound at the close proximity of the LPS binding site. Upon binding, the interaction of vitexin with TLR4/MD-2 complex will, later, cause a disturbance to LPS to interact with the amino acids located at the binding site of TLR4/MD-2 complex. However, such situation can only be happening only if the compound is given in pre-treatment manner. Conversely, donepezil managed to bind at Ile124 and Tyr131 residues of the binding site of TLR4/MD-2 complex. This translates that donepezil can potentially inhibit the binding of LPS at TLR4/MD-2 complex and hence, prevent the activation of microglia and eventually reducing the amount of pro-inflammatory cytokines being produced.

Upon performing MD simulation, the study found that both donepezil- and vitexin-TLR4/MD-2 complexes managed to stably bind throughout the course of 100 ns. Interestingly, vitexin-TLR4/MD-2 complex was able to stabilise much longer with less fluctuations when compared with the donepezil-TLR4/MD-2 complex. This suggests that donepezil-TLR4/MD-2 complex had undergone structural transition.

The present study was focused only on molecular docking and molecular dynamics of vitexin with the aims to explore the ability of the compound to bind at the binding site of TLR4/MD-2 complex and remain stabilised for the course of 100 ns. As such, a number of limitations for the present study can be noted in which the molecular mechanics Poisson-Boltzmann surface area (MM-PBSA) was not been addressed. Also, future study should consider to explore the ability of the compound to penetrate the blood-brain barrier (BBB) to provide more comprehensive information on the potential of vitexin as an antagonist against LPS.

## Conclusion

The results from the present study revealed that vitexin has the potential to act as an antagonist for LPS in the activation of microglia in which the binding energy in MM-GBSA for vitexin is found higher than in donepezil. The hindrance of LPS to bind at the binding site of TLR4/MD-2 complex will prevent the activation of microglia and thus, preventing the over production of pro-inflammatory cytokines which eventually allowing the neurons to thrive. On the other hand, the present study also provides a new insight on the ability of donepezil to interact with TLR4/MD-2 complex. Though donepezil managed to bind at two exact amino acids as LPS at TLR4/MD-2 complex, however, donepezil-TLR4/MD-2 complex showed noticeable fluctuations in comparison with vitexin-TLR4/MD-2 complex. In addition, the consumption of donepezil as part of Alzheimer’s disease treatment can only delay the progress of the disease, however, it does not cure the disease. Hence, a new antagonist is much needed to overcome the situation in parallel with therapeutic strategy to inhibit the attachment of LPS at TLR4/MD-2 complex.

## Data Availability

The datasets generated and/or analysed during the current study are not publicly available as the authors are currently using the datasets for the next study. However, the datasets are available from the corresponding author upon reasonable request.
